# Impact of mask wearing time on fluid consumption and physical activity during the COVID-19 pandemic

**DOI:** 10.3389/fnut.2024.1517702

**Published:** 2025-01-07

**Authors:** Mitchell E. Zaplatosch, Cory L. Butts, Samantha E. Scarneo-Miller, William M. Adams

**Affiliations:** ^1^Department of Exercise Science and Sport Management, Kennesaw State University, Kennesaw, GA, United States; ^2^Department of Kinesiology, University of North Carolina at Greensboro, Greensboro, NC, United States; ^3^Department of Health, Human Performance and Recreation, University of Arkansas, Fayetteville, AR, United States; ^4^Division of Athletic Training, School of Medicine, West Virginia University, Morgantown, WV, United States; ^5^Adams Sports Medicine Consulting LLC, Colorado Springs, CO, United States; ^6^School of Sport, Exercise and Health Sciences, National Centre for Sport and Exercise Medicine (NCSEM), Loughborough University, Loughborough, United Kingdom; ^7^Department of Orthopaedics, University of Utah, Salt Lake City, UT, United States

**Keywords:** fluid intake, health behaviors, hydration, exercise, COVID-19, SARS-CoV-2, water

## Abstract

**Purpose:**

To examine the associations between mask-wearing on fluid consumption and physical activity behaviors during the COVID-19 pandemic.

**Methods:**

137 college students (female, 72.5%; age, 26 ± 9 y) completed a survey detailing their fluid intake, physical activity behaviors, and time spent wearing a mask throughout the day during the previous month in the Fall 2020 academic semester.

**Results:**

Increased daily mask wearing time was not associated with total fluid intake (*p* > 0.05). Participants had greater odds of being ‘somewhat active’ compared to ‘inactive’ with an increase in mask wearing time (OR = 1.23 [1.03, 1.47], *p* = 0.022).

**Conclusion:**

Wearing a mask during the COVID-19 pandemic did not influence fluid intake behaviors, however, it increased the likelihood of reported greater levels of physical activity. These factors may be related to an individual being more likely to globally adopt healthier behaviors, however, this needs further exploration.

## Introduction

It is well known that individuals struggle to achieve fluid intake and physical activity guidelines with only 40–60% of males and females, respectively, currently meeting fluid intake guidelines (1), and only 24.2% of individuals following current physical activity guidelines (2). The impact of not meeting the recommended guidelines for these two health behaviors has been associated with increased risk of chronic disease and early morbidity and mortality (3, 4). Within the context of the SARS-CoV-2 (COVID-19) global pandemic, inadequate consumption of fluids (5, 6), and not participating in regular physical activity (7, 8) increased the risk of having poorer survival outcomes once infected with the COVID-19 virus.

Early policy changes implemented in response to the COVID-19 pandemic included requiring the use of masks/face coverings (hereby referred to as *mask*(*s*)) in situations and settings where social distancing was not possible to help prevent the transmission of COVID-19 (9, 10). While seemingly effective in reducing viral transmission (10, 11), such policies may also have the potential to make certain health behaviors, such as meeting daily fluid intake and physical activity guidelines more difficult to achieve. However, any effects of mask wearing on physical outcomes, such as physiological responses to exercise (12, 13), have been shown to be negligible despite some perceived discomfort from wearing masks (14). Conversely, COVID-19-related disruptions such as suspension of in-person classes or changes in habitation among college students were associated with greater alcohol intake (15).

While previous work explored the impact of the COVID-19 pandemic on health-related behaviors, the impact of COVID-19 pandemic societal policy changes (i.e., mask requirements) on health-related behaviors remains largely unexplored. Thus, this cross-sectional study examined the associations between mask wearing on fluid intake and self-reported physical activity behaviors among college students during the COVID-19 pandemic. It was hypothesized that mask requirements would reduce daily fluid intake volumes and physical activity levels in college students.

## Methods

This study was a continuation of our initial work (15) exploring fluid intake behaviors among college students during the initial response to the COVID-19 global pandemic (Spring 2020) (15). Participants completing the initial online survey (*n* = 1,014) were recruited via convenience sampling to participate in a follow-up online survey (Qualtrics, Provo, UT, USA) investigating their daily fluid intake and physical activity behaviors that was distributed in Fall 2020 (November – December 2020). Eligible participants were college students enrolled in college/university in the United States during the Fall 2020 academic semester. This study was ruled exempt from review by the Institutional Review Board at the University of North Carolina at Greensboro (#20–0442), and was in alignment with the Declaration of Helsinki. Consent was obtained by all participants by selecting the “I Agree to Participate” following reading an informational statement provided on the initial page of the online survey.

### Survey tool

The survey (Supplementary File) consisted of four main parts: (1) participant characteristics and demographics, (2) fluid intake behaviors and (3) self-reported physical activity behaviors.

### Participant characteristics and demographics

Participants indicated their sex, race and ethnicity, academic classification (i.e., Freshman, Sophomore, Junior, Senior, Graduate Student), habitation during the 2020–2021 academic year, change in habitation because of COVID-19, whether their university suspended in-person classes, and the state in which the participant’s college or university was located. Participants were also asked the average time they wear a mask each day (Mask Time, “0–1 h,” “1–2 h,” “2–3 h,” “3–4 h,” “5–6 h,” “6–7 h,” “>8 h”). Mask Time was included as a continuous variable in regression models, with the beta coefficient for this variable representing the effect of increasing mask wearing time by 1 h on each outcome variable.

### Habitual fluid intake

To assess fluid intake behaviors participants completed the BEVQ-15 fluid intake questionnaire, which has demonstrated excellent reliability (Cronbach’s *α* = 0.97–0.99) and validity compared to the longer BEVQ-19 (16). Participants were instructed to indicate the types of beverages they consumed on average during the previous month (i.e., 30 days). Beverage types included water, beer, wine, hard liquor, coffee, energy drinks, milk, low fat milk, nut milk, fruit juice, sweetened juice beverages, soft drinks, diet soft drinks, sweet tea, and any additional beverages not included on the survey (“other”). For each beverage type, participants selected an option for how often (“never or less than 1 per week,” “1 time per week,” “2–3 times per week,” “4–6 times per week,” “1 time per day,” “2 times per day,” and “3+ times per day”) and how much each time (“less than 6 fl oz. [3/4 cup],” “8 fl oz. [1 cup],” “12 fl oz. [1.5 cups],” “16 fl oz. [2 cups],” “20 fl oz. [2.5 cups],” “>20 fl oz”). Fluid intake volume for each beverage type and total fluid intake were calculated based on methods previously established (16).

### Physical activity

Participants were instructed to self-report their levels of physical activity during the 7 days prior to completing the survey. To quantify amount of physical activity, participants completed the IPAQ short form questionnaire, which has demonstrated good reliability (Spearman’s *ρ* = 0.8) and validity comparable to other self-reported physical activity surveys (Spearman’s *ρ* = 0.3) (17). This self-reported physical activity questionnaire was used to classify participants as ‘active’, ‘minimally active’, or ‘inactive’ based on current physical activity recommendations by the American College of Sports Medicine (18).

### Statistical analysis

Multiple regression analyses assessed the impact of mask wearing time (Mask Time - continuous) on fluid intake (continuous) from each beverage category, controlling for sex, academic classification, and MET minutes. A separate Poisson regression was run to assess the impact of mask wearing length of a variety of time lengths on the binary outcome variable of meeting or not meeting current fluid intake recommendations [consuming at least 2.5 L or 2 L in males and females, respectively, based on the European Food Safety Authority fluid intake recommendations (19)], with sex and academic classification included as covariates in the model. A separate multinomial logistic regression was used to assess the odds of being classified as ‘active’, ‘minimally active’ or ‘inactive’ based on the IPAQ questionnaire. All data were analyzed using R statistical software (20). Robust standard errors were calculated for the Poisson regression using the ‘sandwich’ package in R (v. 4.3.1). Results tables were constructed using the R package “stargazer” (21).

## Results

One hundred thirty-seven participants (13.5% [137/1014] response rate) completed the follow-up online survey. Subject demographic characteristics are described in Table 1. In brief, our sample was predominately Caucasian (82.5%), female (75.2%), of higher Academic Classification (senior or graduate students, 80.3%), and typically lived off campus (58.4%). Participants most often wore a mask for 0–1 h (25%), followed by 1–2 h (24%), 3–4 h (24%), 5–6 h (15%), 7–8 h (10%), and > 8 h (1%).

**Table 1:** Demographic characteristics of participants.

	Overall (*N* = 137)
Sex	
Female	103 (75.2%)
Male	33 (24.1%)
Prefer not to answer	1 (0.7%)
Age	
Mean (SD)	26 (9)
Median [Min, Max]	23 (19, 56)
Missing	18 (13.1%)
Race	
American Indian	2 (1.5%)
American Indian or Alaskan Native and White or Caucasian	1 (0.7%)
American Indian or Alaskan Native, White or Caucasian, and Black or African American	1 (0.7%)
Asian	7 (5.1%)
Black or African American	11 (8.0%)
Other	2 (1.5%)
White or Caucasian	113 (82.5%)
Ethnicity	
Hispanic or Latino Origin	9 (6.6%)
Not Hispanic or Latino or Spanish Origin	128 (93.4%)
Academic classification	
Graduate Student	68 (49.6%)
Junior	22 (16.1%)
Senior	42 (30.7%)
Sophomore	4 (2.9%)
Missing	1 (0.7%)
Physical activity classification	
Active	11 (8.0%)
Inactive	58 (42.3%)
Minimally active	52 (38.0%)
Missing	16 (11.7%)
Living arrangements pre-COVID	
Off campus	14 (10.2%)
On campus	33 (24.1%)
Missing	90 (65.7%)
Current living arrangements	
Off campus	80 (58.4%)
On campus	14 (10.2%)
Other	4 (2.9%)
Parent/guardian	17 (12.4%)
Spouse/partner	22 (16.1%)
Living change?	
No	90 (65.7%)
Yes	47 (34.3%)
Masks required?	
No	1 (0.7%)
Yes	134 (97.8%)
Missing	2 (1.5%)

### Influence of mask wearing time on fluid intake behaviors

Greater Mask Time was not associated with total fluid intake (*p* = 0.86) (Tables 2, 3). Female participants reported consuming significantly less total fluid than males (*β* = −376 mL [−687, −65], *p* = 0.018). Greater Mask Time was independently associated with increased sweetened juice beverage consumption (*β* = 9.7, [1.5, 17.9] *p* = 0.02; Table 3), but had no relation to intake of other beverages (*p* > 0.05, Table 3).

**Table 2:** Distribution of total fluid intake and physical activity classification by each category (duration of mask wearing time) of mask time.

Mask time	0–1 h (*N* = 28)	1–2 h (*N* = 31)	3–4 h (*N* = 26)	5–6 h (*N* = 19)	7–8 h (*N* = 11)
Total fluid (mL)					
Median [Q1, Q3]	1567.40 [1159.28, 1960.73]	1910.45 [1413.61, 2283.08]	1662.03 [1403.26, 2266.81]	1798.07 [1378.13, 2398.41]	2058.32 [1780.33, 2407.29]
PA classification					
Active	2 (7.1%)	3 (9.7%)	2 (7.7%)	2 (10.5%)	1 (9.1%)
Inactive	17 (60.7%)	13 (41.9%)	7 (26.9%)	2 (10.5%)	6 (54.5%)
Minimally active	5 (17.9%)	12 (38.7%)	13 (50.0%)	12 (63.2%)	3 (27.3%)
Missing	4 (14.3%)	3 (9.7%)	4 (15.4%)	3 (15.8%)	1 (9.1%)

**Table 3:** Multiple regression analyses assessing the effect of mask time on fluid intake behaviors, accounting for academic classification (continuous 0 = Freshman, 4 = graduate student), Sex (0 = Male, 1 = Female), and MET-minutes of activity.

	Dependent variable								
	Total fluid	Water	Milk	Low fat milk	Nut milk	Fruit juice	Sweetened juice beverage	Wine	Hard liquor
	(1)	(2)	(3)	(4)	(5)	(6)	(7)	(8)	(9)
Intercept	1,568.0^***^ (880.9, 2,255.1)	1,022.9^***^ (578.3, 1,467.5)	72.8 (−36.8, 182.4)	−18.9 (−79.3, 41.4)	113.8^*^ (18.8, 208.7)	133.7^**^ (50.9, 216.4)	143.6^**^ (52.3, 235.0)	−9.6 (−65.2, 46.0)	9.3 (−18.2, 36.8)
MET minutes	0.001 (−0.01, 0.01)	0.003 (−0.01, 0.01)	0.001 (−0.001, 0.003)	−0.000 (−0.001, 0.001)	0.001 (−0.001, 0.002)	−0.000 (−0.002, 0.001)	−0.001 (−0.002, 0.001)	−0.000 (−0.001, 0.001)	−0.000 (−0.001, 0.000)
Sex	−376.1^*^ (−684.3, −67.9)	−91.6 (−291.1, 107.8)	−63.2^*^ (−112.4, −14.1)	21.4 (−5.7, 48.4)	−17.8 (−60.4, 24.8)	−20.2 (−57.4, 16.9)	−33.8 (−74.8, 7.2)	−2.1 (−27.0, 22.9)	−2.7 (−15.1, 9.6)
Mask time	5.5 (−55.6, 66.6)	−15.0 (−54.6, 24.5)	−1.2 (−11.0, 8.5)	2.6 (−2.7, 8.0)	−0.8 (−9.2, 7.7)	5.7 (−1.6, 13.1)	9.7^*^ (1.5, 17.8)	−1.2 (−6.1, 3.8)	−0.3 (−2.8, 2.1)
Academic classification	186.2^*^ (13.8, 358.6)	14.4 (−97.1, 126.0)	11.0 (−16.5, 38.5)	8.5 (−6.7, 23.6)	−13.5 (−37.3, 10.4)	−26.6^*^ (−47.3, −5.8)	−30.8^**^ (−53.7, −7.8)	14.7^*^ (0.7, 28.6)	2.9 (−4.0, 9.8)
Observations	133	133	133	133	133	133	133	133	133
*R* ^2^	0.1	0.02	0.1	0.03	0.02	0.1	0.1	0.04	0.01
Adjusted *R*^2^	0.1	−0.02	0.04	0.002	−0.01	0.03	0.1	0.01	−0.02
Residual Std. Error (df = 128)	828.7	536.3	132.2	72.8	114.5	99.8	110.2	67.1	33.2
*F* Statistic (df = 4; 128)	3.3^*^	0.5	2.3	1.1	0.6	2.2	3.4^*^	1.2	0.3

	Dependent variable							
	Beer	Coffee	Coffee with Cream/Milk	Energy drinks	Soft drinks	Diet soft drinks	Sweet tea	Other
	(1)	(2)	(3)	(4)	(5)	(6)	(7)	(8)
Intercept	0.3 (−2.0, 2.6)	−93.0 (−342.1, 156.2)	23.0 (−176.7, 222.7)	71.7 (−65.2, 208.5)	96.1 (−13.3, 205.5)	−44.4 (−207.8, 119.0)	53.8 (−6.3, 113.9)	−6.8 (−126.2, 112.7)
MET minutes	−0.000 (−0.000, 0.000)	−0.000 (−0.005, 0.005)	−0.000 (−0.004, 0.003)	−0.000 (−0.003, 0.002)	−0.001 (−0.003, 0.001)	−0.001 (−0.004, 0.003)	−0.000 (−0.001, 0.001)	−0.000 (−0.002, 0.002)
Sex	−1.4^**^ (−2.4, −0.4)	−54.9 (−166.7, 56.9)	7.6 (−82.0, 97.1)	−38.1 (−99.6, 23.3)	−41.6 (−90.7, 7.5)	−23.2 (−96.6, 50.1)	−5.7 (−32.6, 21.3)	−9.9 (−63.5, 43.7)
Mask time	0.03 (−0.2, 0.2)	21.2 (−0.9, 43.4)	−16.8 (−34.6, 0.9)	4.8 (−7.4, 16.9)	−2.5 (−12.3, 7.2)	−0.5 (−15.1, 14.0)	3.1 (−2.2, 8.5)	−3.2 (−13.8, 7.4)
Academic classification	0.8^*^ (0.2, 1.3)	96.3^**^ (33.8, 158.8)	64.5^*^ (14.4, 114.6)	−0.2 (−34.6, 34.1)	1.7 (−25.7, 29.2)	39.8 (−1.2, 80.8)	−11.7 (−26.8, 3.4)	15.2 (−14.7, 45.2)
Observations	133	133	133	133	133	133	133	133
*R* ^2^	0.1	0.1	0.1	0.02	0.03	0.04	0.03	0.01
Adjusted *R*^2^	0.1	0.1	0.04	−0.01	−0.005	0.01	−0.003	−0.02
Residual Std. Error (df = 128)	2.8	300.5	240.9	165.1	131.9	197.1	72.5	144.1
F Statistic (df = 4; 128)	4.6^**^	4.2^**^	2.3	0.6	0.8	1.2	0.9	0.4

^*^p < 0.05, ^**^p < 0.01, ^***^p < 0.001.

On average, participants consumed 1924 ± 859 mL of fluid. Mean fluid intake was below current recommendations based on both the Institute of Medicine (22) and European Food Safety Authority Guidelines (19) (males, 2,273 ± 846 mL; females, 1820 ± 838 mL). Specifically, eighty-seven participants (64%) were classified as not meeting fluid intake recommendations (i.e., low drinkers), while forty-nine participants (36%) were classified as “high drinkers.” When including sex and academic classification as covariates, Mask Time (*β* = 0.067 ± 0.048, *p* = 0.162) did not influence one’s ability to meet daily fluid intake recommendations. Females consumed significantly less Milk, and Beer compared to males, when controlling for Mask Time, Academic Classification, and MET minutes of physical activity (Table 3). Being of higher Academic Classification was independently associated with increased total fluid intake and greater intake of wine, beer, coffee (with and without cream), and decreased consumption of fruit juice and sweetened juice beverages.

### Influence of mask wearing time on physical activity behaviors

Participants had greater odds of being “minimally active” compared to “inactive” as Mask Time increased (OR = 1.24 [1.01, 1.50], *p* = 0.035, Table 4), when controlling for Sex and Academic Classification. However, Mask Time was not associated with odds of being in the “active” category compared to “inactive” (OR = 1.12 [0.82, 1.54], *p* = 0.50, Table 4). Academic Classification and Sex did not influence odds of being in a higher activity category (*p* > 0.05).

**Table 4:** Odds of being “Minimally Active” or “Active” based on IPAQ classifications compared to “Inactive,” when controlling for sex, age, and academic classification.

Variable	OR^1^	95% CI^1^	*p*-value
Active			
Mask Time	1.23	[0.93, 1.63]	0.200
Sex			
Female	—	—	
Male	2.50	[0.63, 9.90]	0.200
Academic classification	0.90	[0.40, 2.00]	0.800
Minimally active			
Mask time	1.23	[1.03, 1.47]	0.022
Sex			
Female	—	—	
Male	0.64	[0.24, 1.72]	0.40
Academic classification	0.83	[0.52, 1.32]	0.40

^1OR = Odds ratio, CI = Confidence interval.^

Most participants were “inactive” (42.3%) or “minimally active” (38.0%) based on the IPAQ physical activity classifications. One participant selected “prefer not to answer” for the question asking participant’s sex; this participant was excluded from analyses including “sex” as an independent variable.

## Discussion

This study characterized the associations between the use of masks on fluid intake and self-reported physical activity among college students during the Fall 2020 academic semester. A majority (64%) of participants were not meeting fluid intake recommendations, were largely inactive (42.3%) or minimally active (38.0%) and tended to wear masks between 0 and 3 h each day. Although our results suggest mask wearing is not associated with differences in total fluid intake as a continuous variable or the number of individuals meeting fluid intake recommendations, it may be that since 49% of our participants wore a mask less than 3 h per day, there was limited impact on the ease of consuming fluids. Further, we found greater odds of falling into the ‘minimally active’ physical activity category with increasing time spent wearing a mask, which suggests that the mask wearing policies that were put in place in response to the COVID-19 pandemic may not reduce physical activity participation. However, since individuals were not surveyed prior to the COVID-19 pandemic and the associated implementation of mask policies, this is speculative and there may be other psychosocial factors that influenced one’s propensity to have adopted the health behaviors assessed in this study (i.e., fluid intake, physical activity, mask wear time). By contrast, wearing a mask may offer additional perceived safety for those looking to participate in at least some physical activity during a pandemic compared to sedentary individuals.

Although most (87/137) of our participants did not meet fluid intake recommendations, this was unrelated to mask wearing. Previous research has identified barriers to hydration as an important determinant of fluid intake (23), but our data suggest mask wearing time does not serve as an additional barrier impeding fluid consumption. Instead, it is likely the other factors (e.g., accessibility of fluid and concentrated efforts to meet fluid consumption goals) (24) are more important for this behavior.

Reported fluid intake among our participants was lower than those reported by Sahin et al. who examined fluid intake in young adults during the COVID-19 pandemic in Turkey (25) and found 74.5% of males and 93.5% of females met water intake recommendations. By contrast, 64% of our sample did *not* meet fluid intake recommendations. Given the diversity of fluid intake practices that has been noted between different countries (1, 24, 26–29), cultural differences in fluid intake patterns should also be considered within the context of the pandemic. Further, differences in the enactment, enforcement, and adherence to mask mandates between countries should be considered when examining the influence of these mandates on health behaviors such as fluid intake.

In our sample, longer time spent wearing masks was associated with higher odds of being minimally active compared to the inactive reference category. From these data, the rationale for this relationship is unclear, but perhaps individuals who wore masks were more likely to feel protected from viral transmission (30) and able to engage in some physical activity around others. Furthermore, individual perceptions regarding the efficacy of masks for preventing COVID-19 may have influenced our results. It is plausible that individuals who are generally more health conscious are more likely to take additional protective measures to reduce the risk of illness, while maintaining other beneficial health behaviors such as adequate fluid intake, physical activity, and vaccination (31). However, if this were true, we would have expected an association between longer mask wearing length and odds of being in the “Active” category.

Regarding physical activity levels, isolation/quarantine orders were still common at the time of survey administration (November – December 2020). Such isolation periods have the potential to reduce engagement in physical activity if one’s preferred form of activity tends to involve social engagement (32) (i.e., group fitness classes, exercising with friends in a gym environment, etc.), and high social support was associated with 64% increased odds of maintaining physical activity during isolation periods (33). Reported physical activity in the present study was consistent with previous research suggesting low physical activity in college students, regardless of the pandemic (34–38). A recent scoping review identified a decrease in physical activity related to social distancing measures, despite the potential for physical activity to reduce the increased mental health burden associated with the pandemic (35).

Specific to college students, a recent study among students living in Spain observed a decline in physical activity in conjunction with increased sitting time during lockdown (36). Another study conducted in the U.S. before and after the 2020 spring break season, during which many universities transitioned from standard teaching methods to virtual learning, saw a significant reduction in step count (37). Sidebottom et al. (38) conducted a similar cross-sectional survey in college students, finding decreased physical activity and increased sedentary behavior pre- and during the COVID-19 quarantine period. Our data, in addition to these prior studies, illustrate the impact the COVID-19 pandemic and associated mitigation strategies have had on physical activity participation among college-age adults. Specifically, mask wearing may enable participation in physical activity in those who might have otherwise been inactive, perhaps due to perceived risk of COVID-19 infection. Together, these findings will inform future efforts to mitigate infection transmission without concern that protective measures such as mask wearing will limit fluid intake or physical activity. The massive scale of the pandemic and its aftermath warrant continued investigation into such strategies such that other health promoting behaviors are not inhibited in an attempt to reduce viral transmission.

### Limitations

Given the cross-sectional nature of our study, we are not able to draw inferences regarding the direct impact of time spent wearing a mask on fluid intake outcomes. Furthermore, a large percentage of our sample had a short mask wear time (49% wore a mask <2 h), likely due to a transition to virtual classes for many universities. However, such behaviors could also be due to limited adherence to mask mandates, as has been previously reported (39). Further research should consider the impact of compliance with masks and other COVID-19 related mandates on other health behaviors. We also did not capture where participants typically complete physical activity (i.e., walking outside versus exercising in a gym), which may have confounded the null relationship between physical activity, mask wearing length, and fluid intake behaviors when including in the same model. Future research should consider differences in fluid intake behaviors within versus outside of a gym setting, to determine the impact of perceived environmental risk on fluid intake behaviors. Our survey of fluid intake and physical activity were also based on slightly different timescales (previous month compared to the previous 7 days).

It was also beyond the scope of the present study to assess individual beliefs influencing one’s propensity toward engaging in physical activity or drinking fluid, and how an additional perceived barrier such as wearing a mask impacts the likelihood of engaging in these behaviors through this mechanism. Additional study should assess how the COVID-19 pandemic and associated policy changes have influenced the likelihood of adopting this and other health behaviors, in accordance with components of the health belief model (31). Our survey was also conducted prior to the widespread availability of vaccines. Given ever-changing policies and regional differences in mask mandates both at the state and local government level, there may be variability in fluid intake, physical activity, and mask wearing habits specific to each individual region.

## Conclusion

Daily mask wearing time during the COVID-19 pandemic was not associated with fluid intake behaviors in college students but was associated with greater odds of being somewhat active compared to inactive during the COVID-19 pandemic. Further research efforts should target the underlying factors and perceptions that influence one’s decision to engage in these health behaviors regardless of the presence of a global pandemic. With such low participation in physical activity nationwide, attempts should be made to ensure that any policy changes that have the potential to inhibit physical activity are offset by ways to encourage physical activity or other health behaviors.

## Data Availability

The raw data supporting the conclusions of this article will be made available by the authors, without undue reservation.
