# Supplementary material for: Impact of mask wearing time on fluid consumption and physical activity during the COVID-19 pandemic
Source: Front Nutr. 2025 Jan 7;11:1517702. doi: 10.3389/fnut.2024.1517702 (PMC11752909; doi:10.3389/fnut.2024.1517702)
Supplement: Supplementary file 1 [file Data_Sheet_1.PDF]

Q1

What is your age?

Q5

What is your sex?

- Male
- Female
- Prefer not to answer

Q3

What is your ethnicity?

- Hispanic or Latino or Spanish Origin
- Not Hispanic or Latino or Spanish Origin

Q4

What is your race (select all that apply)?

- American Indian or Alaskan Native
- Asian
- Black or African American
- Native Hawaiian or other Pacific Islander
- White or Caucasian
- Other

Q97

What state do you currently live?

Alabama

Q78

What is the zip code in which you live?

Q71

What state is your University/College Located

Alabama

Q79

What is the zip code of the University/College in which you attend?

Q9

For the 2020-2021 academic year, please select the option that best defines your living arrangements.

Lived on campus

Q75

Please select the instruction format your classes for the Fall 2020 semester. Select all that apply

- In Person/Face to Face
- Online
- Hybrid (mix of online and in person instruction)
- Hands on laboratory course
- Online laboratory course

Q76

Does your University/College require you to wear a mask or face covering when you are on campus?

- Yes
- No

Q77

On average, how many hours throughout the day do you wear a mask or face covering?

- 1 - 2 hours
- 3 - 4 hours
- 5 - 6 hours
- 7 - 8 hours
- > 8 hours

Q68

Did your University/College suspend face-to-face courses during the Spring 2020 semester due to the COVID-19 Pandemic?

- Yes
- No

Q74

During the Spring 2020 Semester, please select the option that best defined your living arrangement

Lived on campus

Q69

Did your living arrangements during the 2019-2020 academic year change as a result of the COVID-19 pandemic?

- Yes
- No

Condition: Yes Is Selected. Skip To: Please indicate what your living arra....

Condition: No Is Selected. Skip To: What defines your current academic st....

Q70

Please indicate what your living arrangements were prior to the COVID-19 pandemic

Live on campus

Q2

What defines your current academic standing?

Freshman

BEVQ-15 Block Options

Q6

<u>Instructions:</u> For the past month, please indicate your intake for each beverage type by marking the appropriate selection for "how often" and "how much each time".

1. Indicate how often you drank the following beverages, for example, if you drank 5 glasses of water per week, mark 4-6 times per week.

2. Indicate the approximate amount of beverage you drank each time, for example, if you drank 1 cup of water each time, mark 1 cup under "how much each time". If applicable, indicate the specific type of beverage by marking an "X" in the bubble by the one used (i.e., type of nut milk).

3. When trying to estimate your intake throughout the day, (i.e., water) think about the total amount you drink. For example, 3 times per day and 20 fl oz each time = 60 fl oz per day. If you consume more 60 fl oz per day select "1 time per day" and write the TOTAL daily amount in the last column.

4. Do not count beverages used in cooking or other preparations, such as milk in cereal.

5. Count milk/creamer added to tea and coffee in the tea or coffee with creamer beverage category, NOT in the milk categories; this includes non-dairy creamer. Please indicate the type of creamer (flavored, plain or sugar-free) and sweetener used by marking an "X" in the bubble by the one used, if applicable.

Q7

How often did you consume **<u>Water</u>** within the past month?

Never or less than 1 time per week	1 time per week	2-3 times per week	4-6 times per week	1 time per day	2 times per day	3+ times per day
○	○	○	○	○	○	○

Q8

How much **<u>Water</u>** did you consume each time?

Less than 6 fl oz (3/4 cup)	8 fl oz (1 cup)	12 fl oz (1 1/2 cups)	16 fl oz (2 cups)	20 fl oz (2 1/2 cups)	>20 fl oz
○	○	○	○	○	○

Condition: >20 fl oz Is Selected. Skip To: Please specify how much Water in flui....

Condition: >20 fl oz Is Not Selected. Skip To: How often did you consume 100% Fruit ....

Q10

Please specify how much **<u>Water</u>** in fluid ounces you consumed each day

21

Q11

How often did you consume **100% Fruit Juice** within the past month?

Never or less than 1 time per week	1 time per week	2-3 times per week	4-6 times per week	1 time per day	2 times per day	3+ times per day
○	○	○	○	○	○	○

Q12

How much **100% Fruit Juice** did you consume each time?

Less than 6 fl oz (3/4 cup)	8 fl oz (1 cup)	12 fl oz (1 1/2 cups)	16 fl oz (2 cups)	20 fl oz (2 1/2 cups)	>20 fl oz
○	○	○	○	○	○

Condition: >20 fl oz Is Selected. Skip To: Please specify how much 100% Fruit Ju....

Condition: >20 fl oz Is Not Selected. Skip To: How often did you consume Sweetened J....

Q13

Please specify how much **100% Fruit Juice** in fluid ounces you consumed each day

21

Q14

How often did you consume **Sweetened Juice Beverage/Drink** (fruit punch, juice cocktail, Sunny Delight, Capri Sun) within the past month?

Never or less than 1 time per week	1 time per week	2-3 times per week	4-6 times per week	1 time per day	2 times per day	3+ times per day
○	○	○	○	○	○	○

Q15

How much **Sweetened Juice Beverage/Drink**(fruit punch, juice cocktail, Sunny Delight, Capri Sun) did you consume each time?

Less than 6 fl oz (3/4 cup)	8 fl oz (1 cup)	12 fl oz (1 1/2 cups)	16 fl oz (2 cups)	20 fl oz (2 1/2 cups)	>20 fl oz
○	○	○	○	○	○

Condition: >20 fl oz Is Selected. Skip To: Please specify how much Sweetened Jui....

Condition: >20 fl oz Is Not Selected. Skip To: How often did you consume Whole Milk ....

Q16

Please specify how much **Sweetened Juice Beverage/Drink**(fruit punch, juice cocktail, Sunny Delight, Capri Sun) in fluid ounces you consumed each day

21

Q17

How often did you consume **Whole Milk** (red cap), **2% Milk** (purple cap), or **Chocolate Milk** within the past month?

Never or less than 1 time per week	1 time per week	2-3 times per week	4-6 times per week	1 time per day	2 times per day	3+ times per day
○	○	○	○	○	○	○

Q18

How much **Whole Milk**(red cap), **2% Milk**(purple cap), or **Chocolate Milk** did you consume each time?

Less than 6 fl oz (3/4 cup)	8 fl oz (1 cup)	12 fl oz (1 1/2 cups)	16 fl oz (2 cups)	20 fl oz (2 1/2 cups)	>20 fl oz
○	○	○	○	○	○

Condition: >20 fl oz Is Selected. Skip To: Please specify how much Whole Milk(re....

Condition: >20 fl oz Is Not Selected. Skip To: How often did you consume Low Fat 1% ....

Q19

Please specify how much **Whole Milk**(red cap), **2% Milk**(purple cap), or **Chocolate Milk** in fluid ounces you consumed each day

21

Q20

How often did you consume **Low Fat 1% Milk** (green cap), **Fat Free/Skim Milk** (light blue cap), **Buttermilk or Soy Milk** within the past month?

Never or less than 1 time per week	1 time per week	2-3 times per week	4-6 times per week	1 time per day	2 times per day	3+ times per day
○	○	○	○	○	○	○

Q21

How much **Low Fat 1% Milk**(green cap), **Fat Free/Skim Milk**(light blue cap), **Buttermilk or Soy Milk** did you consume each time?

Less than 6 fl oz (3/4 cup)	8 fl oz (1 cup)	12 fl oz (1 1/2 cups)	16 fl oz (2 cups)	20 fl oz (2 1/2 cups)	>20 fl oz
○	○	○	○	○	○

Condition: >20 fl oz Is Selected. Skip To: Please specify how much Low Fat 1% Mi....

Condition: >20 fl oz Is Not Selected. Skip To: How often did you consume Nut Milk (a....

Q22

Please specify how much **Low Fat 1% Milk**(green cap), **Fat Free/Skim Milk**(light blue cap), **Buttermilk or Soy Milk** in fluid ounces you consumed each day

21

Q23

How often did you consume **Nut Milk** (almond, cashew, coconut) within the past month?

Never or less than 1 time per week	1 time per week	2-3 times per week	4-6 times per week	1 time per day	2 times per day	3+ times per day
○	○	○	○	○	○	○

Condition: Never or less than 1 time p... Is Not Selected. Skip To: Select the the type of Nut Milk (almo....

Condition: Never or less than 1 time p... Is Selected. Skip To: How much Nut Milk (almond, cashew, co....

Q65

Select the the type of **Nut Milk** (almond, cashew, coconut) you consumed. Please select all that apply

- ○ Flavored, Original, or Plain
- ○ Unsweetened

Q24

How much **Nut Milk** (almond, cashew, coconut) did you consume each time?

Less than 6 fl oz (3/4 cup)	8 fl oz (1 cup)	12 fl oz (1 1/2 cups)	16 fl oz (2 cups)	20 fl oz (2 1/2 cups)	>20 fl oz
○	○	○	○	○	○

Condition: >20 fl oz Is Selected. Skip To: Please specify how much Nut Milk (alm....

Condition: >20 fl oz Is Not Selected. Skip To: How often did you consume Soft Drinks....

Q25

Please specify how much **Nut Milk** (almond, cashew, coconut) in fluid ounces you consumed each day

21

Q26

How often did you consume **Soft Drinks, Regular** within the past month?

Never or less than 1 time per week	1 time per week	2-3 times per week	4-6 times per week	1 time per day	2 times per day	3+ times per day
○	○	○	○	○	○	○

Q27

How much **Soft Drinks, Regular** did you consume each time?

Less than 6 fl oz (3/4 cup)	8 fl oz (1 cup)	12 fl oz (1 1/2 cups)	16 fl oz (2 cups)	20 fl oz (2 1/2 cups)	>20 fl oz
○	○	○	○	○	○

Condition: >20 fl oz Is Selected. Skip To: Please specify how much Soft Drinks, ...

Condition: >20 fl oz Is Not Selected. Skip To: How often did you consume Energy & Sp....

Q28

Please specify how much **Soft Drinks, Regular** in fluid ounces you consumed each day

21

Q29

How often did you consume **Energy & Sports Drinks, Regular** (Red Bull, Gatorade, Powerade) within the past month?

Never or less than 1 time per week	1 time per week	2-3 times per week	4-6 times per week	1 time per day	2 times per day	3+ times per day
○	○	○	○	○	○	○

Q30

How much **Energy & Sports Drinks, Regular** (Red Bull, Gatorade, Powerade) did you consume each time?

Less than 6 fl oz (3/4 cup)	8 fl oz (1 cup)	12 fl oz (1 1/2 cups)	16 fl oz (2 cups)	20 fl oz (2 1/2 cups)	>20 fl oz
○	○	○	○	○	○

Condition: >20 fl oz Is Selected. Skip To: Please specify how much Energy & Spor....

Condition: >20 fl oz Is Not Selected. Skip To: How often did you consume Diet or Art....

Q31

Please specify how much **Energy & Sports Drinks, Regular** (Red Bull, Gatorade, Powerade) in fluid ounces you consumed each day

21

Q32

How often did you consume **Diet or Artificially Sweetened Soft Drinks, Energy & Sports Drinks** (Diet Coke, Crystal Light, Artificially Sweetened Sparkling Water, Sugar-Free or Total Zero Red Bull, Powerade Zero) within the past month?

Never or less than 1 time per week	1 time per week	2-3 times per week	4-6 times per week	1 time per day	2 times per day	3+ times per day
○	○	○	○	○	○	○

Q33

How much **Diet or Artificially Sweetened Soft Drinks, Energy & Sports Drinks** (Diet Coke, Crystal Light, Artificially Sweetened Sparkling Water, Sugar-Free or Total Zero Red Bull, Powerade Zero) did you consume each time?

Less than 6 fl oz (3/4 cup)	8 fl oz (1 cup)	12 fl oz (1 1/2 cups)	16 fl oz (2 cups)	20 fl oz (2 1/2 cups)	>20 fl oz
○	○	○	○	○	○

Condition: >20 fl oz Is Selected. Skip To: Please specify how much Diet or Artif....

Condition: >20 fl oz Is Not Selected. Skip To: How often did you consume Sweet Tea w....

Q34

Please specify how much **Diet or Artificially Sweetened Soft Drinks, Energy & Sports Drinks** (Diet Coke, Crystal Light, Artificially Sweetened Sparkling Water, Sugar-Free or Total Zero Red Bull, Powerade Zero) in fluid ounces you consumed each day

21

Q35

How often did you consume **Sweet Tea with Sugar** within the past month?

Never or less than 1 time per week	1 time per week	2-3 times per week	4-6 times per week	1 time per day	2 times per day	3+ times per day
○	○	○	○	○	○	○

Q36

How much **Sweet Tea with Sugar** did you consume each time?

Less than 6 fl oz (3/4 cup)	8 fl oz (1 cup)	12 fl oz (1 1/2 cups)	16 fl oz (2 cups)	20 fl oz (2 1/2 cups)	>20 fl oz
○	○	○	○	○	○

Condition: >20 fl oz Is Selected. Skip To: Please specify how much Sweet Tea wit....

Condition: >20 fl oz Is Not Selected. Skip To: How often did you consume Tea or Coff....

Q37

Please specify how much **Sweet Tea with Sugar** in fluid ounces you consumed each day

21

Q38

How often did you consume **Tea or Coffee, Black** (no creamer or milk) within the past month?

Never or less than 1 time per week	1 time per week	2-3 times per week	4-6 times per week	1 time per day	2 times per day	3+ times per day
○	○	○	○	○	○	○

Q64

Select the appropriate additions to **Tea or Coffee, Black**. Please select all that apply

- Sugar
- Artificial Sweetener
- Not Applicable

Q39

How much **Tea or Coffee, Black** (no creamer or milk) did you consume each time?

Less than 6 fl oz (3/4 cup)	8 fl oz (1 cup)	12 fl oz (1 1/2 cups)	16 fl oz (2 cups)	20 fl oz (2 1/2 cups)	>20 fl oz
○	○	○	○	○	○

Condition: >20 fl oz Is Selected. Skip To: Please specify how much Tea or Coffee....

Condition: >20 fl oz Is Not Selected. Skip To: How often did you consume Tea or Coff....

Q40

Please specify how much **Tea or Coffee, Black** (no creamer or milk) in fluid ounces you consumed each day

21

Q41

How often did you consume **Tea or Coffee** (with milk &/or creamer) within the past month?

Never or less than 1 time per week	1 time per week	2-3 times per week	4-6 times per week	1 time per day	2 times per day	3+ times per day
○	○	○	○	○	○	○

Q63

Select the appropriate additions to **Tea or Coffee** (with milk &/or creamer). Please select all that apply

- Sugar
- Artificial Sweetener
- Milk
- Half & Half or Cream
- Creamer, Plain
- Creamer, Flavored
- Creamer, Sugar Free
- × Not Applicable

Q42

How much **Tea or Coffee** (with milk &/or creamer) did you consume each time?

Less than 6 fl oz (3/4 cup)	8 fl oz (1 cup)	12 fl oz (1 1/2 cups)	16 fl oz (2 cups)	20 fl oz (2 1/2 cups)	>20 fl oz
○	○	○	○	○	○

Condition: >20 fl oz Is Selected. Skip To: Please specify how much Tea or Coffee....

Condition: >20 fl oz Is Not Selected. Skip To: How often did you consume Wine (red o....

Q43

Please specify how much **Tea or Coffee** (with milk &/or creamer) in fluid ounces you consumed each day

21

Q44

How often did you consume **Wine** (red or white) within the past month?

Never or less than 1 time per week	1 time per week	2-3 times per week	4-6 times per week	1 time per day	2 times per day	3+ times per day
○	○	○	○	○	○	○

Q45

How much **Wine** (red or white) did you consume each time?

Less than 6 fl oz (3/4 cup)	8 fl oz (1 cup)	12 fl oz (1 1/2 cups)	16 fl oz (2 cups)	20 fl oz (2 1/2 cups)	>20 fl oz
○	○	○	○	○	○

Condition: >20 fl oz Is Selected. Skip To: Please specify how much Wine (red or ....

Condition: >20 fl oz Is Not Selected. Skip To: How often did you consume Hard Liquor....

Q46

Please specify how much **Wine** (red or white) in fluid ounces you consumed each day

21

Q47

How often did you consume **Hard Liquor** (vodka, rum, tequila, etc.) within the past month?

Never or less than 1 time per week	1 time per week	2-3 times per week	4-6 times per week	1 time per day	2 times per day	3+ times per day
○	○	○	○	○	○	○

Q48

How much **Hard Liquor** (vodka, rum, tequila, etc.) did you consume each time?

Less than 6 fl oz (3/4 cup)	8 fl oz (1 cup)	12 fl oz (1 1/2 cups)	16 fl oz (2 cups)	20 fl oz (2 1/2 cups)	>20 fl oz
○	○	○	○	○	○

Condition: >20 fl oz Is Selected. Skip To: Please specify how much Hard Liquor (....

Condition: >20 fl oz Is Not Selected. Skip To: How often did you consume Beer, Ales,....

Q49

Please specify how much **Hard Liquor** (vodka, rum, tequila, etc.) in fluid ounces you consumed each day

21

Q50

How often did you consume **Beer, Ales, Wine Coolers, Non Alcoholic or Light Beer** within the past month?

Never or less than 1 time per week	1 time per week	2-3 times per week	4-6 times per week	1 time per day	2 times per day	3+ times per day
○	○	○	○	○	○	○

Q51

How much **Beer, Ales, Wine Coolers, Non Alcoholic or Light Beer** did you consume each time?

Less than 6 fl oz (3/4 cup)	8 fl oz (1 cup)	12 fl oz (1 1/2 cups)	16 fl oz (2 cups)	20 fl oz (2 1/2 cups)	>20 fl oz
○	○	○	○	○	○

Condition: >20 fl oz Is Selected. Skip To: Please specify how much Beer, Ales, W....

Condition: >20 fl oz Is Not Selected. Skip To: Did you consume any other types of be....

Q52

Please specify how much **Beer, Ales, Wine Coolers, Non Alcoholic or Light Beer** in fluid ounces you consumed each day

21

Q56

Did you consume any other types of beverages?

- Yes
- No

Condition: No Is Selected. Skip To: End of Block.

Condition: Yes Is Selected. Skip To: Please list the type(s) of beverages ....

Q62

Please list the type(s) of beverages you consumed

Q53

How often did you consume **Other** within the past month?

Never or less than 1 time per week	1 time per week	2-3 times per week	4-6 times per week	1 time per day	2 times per day	3+ times per day
○	○	○	○	○	○	○

Q54

How much **Other** did you consume each time?

Less than 6 fl oz (3/4 cup)	8 fl oz (1 cup)	12 fl oz (1 1/2 cups)	16 fl oz (2 cups)	20 fl oz (2 1/2 cups)	>20 fl oz
○	○	○	○	○	○

Condition: >20 fl oz Is Selected. Skip To: Please specify how much Other in flui....

Condition: >20 fl oz Is Not Selected. Skip To: End of Block.

Q55

Please specify how much **Other** in fluid ounces you consumed each day

21

IPAQ-Short

Block Options

Q58

We are interested in finding out about the kinds of physical activities that people do as part of their everyday lives. The questions will ask you about the time you spent being physically active in the last 7 days. Please answer each question even if you do not consider yourself to be an active person. Please think about the activities you do at work, as part of your house and yard work, to get from place to place, and in your spare time for recreation, exercise or sport.

Think about all the **vigorous** activities that you did in the **last 7 days**. **Vigorous** physical activities refer to activities that take hard physical effort and make you breathe much harder than normal. Think only about those physical activities that you did for at least 10 minutes at a time

During the **last 7 days**, on how many days did you do vigorous physical activities like heavy lifting, digging, aerobics, or fast bicycling?

0

Condition: 0 Is Not Selected. Skip To: How much time did you usually spend d....

Q81

How much time did you usually spend doing **vigorous** physical activities on one of those days?

Hours per day	0
Minutes per day	0
Don't know/Not Sure (If applicable, type 99 in box)	0
Total	0

Q59

Think about all the **moderate** activities that you did in the **last 7 days**. **Moderate** activities refer to activities that take moderate physical effort and make you breathe somewhat harder than normal. Think only about those physical activities that you did for at least 10 minutes at a time.

During the **last 7 days**, on how many days did you do **moderate** physical activities like carrying light loads, bicycling at a regular pace, or doubles tennis? Do not include walking.

0

Condition: 0 Is Not Selected. Skip To: How much time did you usually spend d....

Condition: 0 Is Selected. Skip To: Think about the time you spent walkin....

Q82

How much time did you usually spend doing **moderate** physical activities on one of those days?

Hours per day	0
Minutes per day	0
Don't know/Not Sure (If applicable, type 99 in box)	0
Total	0

Q85

Think about the time you spent **walking** in the **last 7 days**. This includes at work and at home, walking to travel from place to place, and any other walking that you have done solely for recreation, sport, exercise, or leisure.

During the **last 7 days**, on how many days did you **walk** for at least 10 minutes at a time?

0

Condition: 0 Is Not Selected. Skip To: How much time did you usually spend w....

Condition: 0 Is Selected. Skip To: The last question is about the time y....

Q86

How much time did you usually spend **walking** on one of those days?

Hours per day	0
Minutes per day	0
Don't know/Not Sure (If applicable, type 99 in box)	0
Total	0

Q88

The last question is about the time you spent **sitting** on weekdays during the **last 7 days**. Include time spent at work, at home, while doing course work and during leisure time. This may include time spent sitting at a desk, visiting friends, reading, or sitting or lying down to watch television.

During the last 7 days, how much time did you spend **sitting** on a week day?

Hours per day	0
Minutes per day	0
Don't know/Not Sure (If applicable, type 99 in box)	0
Total	0

PSQI Block Options

Q89

During the past month, when have you usually gone to bed at night?

Q90

During the past month, how long (in minutes) has it usually taken you to fall asleep each night?

Q91

During the past month, when have you usually gotten up in the morning?

Q92

During the past month, how many hours of actual sleep did you get at night? (This may be different than the number of hours you spend in bed)

- Click to write Choice 1
- Click to write Choice 2
- Click to write Choice 3

Q93

Instructions: For each of the remaining questions, check the one best response. Please answer all questions

During the past month, how often have you had trouble sleeping because you...

	Not during the past month	Less than once a week	Once or twice a week	Three or more times per week
...cannot get to sleep within 30 minutes	○	○	○	○
...wake up in the middle of the night or early morning	○	○	○	○
...have to get up to use the bathroom	○	○	○	○
...cannot breathe comfortably	○	○	○	○
...cough or snore loudly	○	○	○	○
...feel too cold	○	○	○	○
...feel too hot	○	○	○	○
...had bad dreams	○	○	○	○
...have pain	○	○	○	○
Other reason(s)	○	○	○	○

Q94

During the past month, how would you rate your overall sleep quality

Very Good	Fairly Good	Fairly Bad	Very Bad
○	○	○	○

Q95

Click to write the question text

	Not during the past month	Less than once a week	Once or twice a week	Three or more times per week
During the past month, how often have you taken medication (prescribed or "over the counter") to help you sleep?	○	○	○	○
During the past month, how often have you had trouble staying awake while driving, eating meals, or engaging in social activity?	○	○	○	○

Q96

During the past month, how much of a problem has it been for you to keep up enough enthusiasm to get things done?

Not a problem at all	Only a very slight problem	Somewhat of a problem	A very big problem
○	○	○	○

Follow-Up

Block Options

Q72

Thank you for your participation!

Add Block

End of Survey

Survey Termination Options...

Qualtrics.com Contact Information Legal
